# Antigenic characterization of the human immunodeficiency virus (HIV-1) envelope glycoprotein precursor incorporated into nanodiscs

**DOI:** 10.1371/journal.pone.0170672

**Published:** 2017-02-02

**Authors:** Kristen C. Witt, Luis Castillo-Menendez, Haitao Ding, Nicole Espy, Shijian Zhang, John C. Kappes, Joseph Sodroski

**Affiliations:** 1 Department of Cancer Immunology & Virology, Dana-Farber Cancer Institute, Department of Microbiology & Immunobiology, Harvard Medical School, Boston, MA, United States of America; 2 Departments of Medicine, University of Alabama at Birmingham, Birmingham, AL, United States of America; 3 Birmingham Veterans Affairs Medical Center, Research Service, Birmingham, AL, United States of America; 4 Department of Immunology & Infectious Diseases, Harvard School of Public Health, Boston, MA, United States of America; Simon Fraser University, CANADA

## Abstract

The entry of human immunodeficiency virus (HIV-1) into host cells is mediated by the viral envelope glycoproteins (Envs), which are derived by the proteolytic cleavage of a trimeric gp160 Env precursor. The mature Env trimer is a major target for entry inhibitors and vaccine-induced neutralizing antibodies. Env interstrain variability, conformational flexibility and heavy glycosylation contribute to evasion of the host immune response, and create challenges for structural characterization and vaccine development. Here we investigate variables associated with reconstitution of the HIV-1 Env precursor into nanodiscs, nanoscale lipid bilayer discs enclosed by membrane scaffolding proteins. We identified detergents, as well as lipids similar in composition to the viral lipidome, that allowed efficient formation of Env-nanodiscs (Env-NDs). Env-NDs were created with the full-length Env precursor and with an Env precursor with the majority of the cytoplasmic tail intact. The self-association of Env-NDs was decreased by glutaraldehyde crosslinking. The Env-NDs exhibited an antigenic profile expected for the HIV-1 Env precursor. Env-NDs were recognized by broadly neutralizing antibodies. Of note, neutralizing antibody epitopes in the gp41 membrane-proximal external region and in the gp120:gp41 interface were well exposed on Env-NDs compared with Env expressed on cell surfaces. Most Env epitopes recognized by non-neutralizing antibodies were masked on the Env-NDs. This antigenic profile was stable for several days, exhibiting a considerably longer half-life than that of Env solubilized in detergents. Negative selection with weak neutralizing antibodies could be used to improve the antigenic profile of the Env-NDs. Finally, we show that lipid adjuvants can be incorporated into Env-NDs. These results indicate that Env-NDs represent a potentially useful platform for investigating the structural, functional and antigenic properties of the HIV-1 Env trimer in a membrane context.

## Introduction

Human immunodeficiency virus (HIV-1) entry into the host cell is mediated by the viral envelope glycoproteins (Envs), which are derived by the proteolytic cleavage of a trimeric gp160 Env precursor [1–3]. The resulting mature Env complex consists of three gp120 exterior subunits and three gp41 transmembrane subunits. The metastable Env trimer is triggered to undergo conformational changes by binding to the receptors, CD4 and CCR5/CXCR4 [1,4–17]. CD4 binding induces the formation of a pre-hairpin intermediate, and CCR5/CXCR4 binding drives the formation of a stable gp41 six-helix bundle required for the fusion of the viral and target cell membranes [4–7,18–24].

The Env trimer is the sole virus-specific target on the virion surface available for the binding of neutralizing antibodies [1–3,25–28]. To allow persistence in the host, HIV-1 Envs have evolved features to minimize the elicitation and impact of broad neutralizing antibodies [1,29,30]. These features include surface variability among HIV-1 strains, conformational lability, and a heavy glycan coat [29–34]. Most anti-Env antibodies elicited during natural HIV-1 infection do not efficiently recognize functional Env spikes, and therefore fail to neutralize the virus [25,29,30,35–38]. Some antibodies are directed against variable regions on the Env surface, and select for HIV-1 variants that escape neutralizing activity of these antibodies [39–43]. Only after several years of infection and in a minority of infected individuals are broadly neutralizing antibodies generated [44–49].

The Env features that minimize the elicitation and effectiveness of virus-suppressive antibodies also create challenges for structural studies. Cryoelectron tomography has provided low-resolution density maps of the mature HIV-1 virion Env spike [50]. Soluble Env trimers lacking the transmembrane anchor and cytoplasmic tail have been produced, and detailed structures of stabilized forms of these trimers have been solved by x-ray crystallography and cryoelectron microscopy [51–54]. Membrane-anchored HIV-1 Envs have been solubilized in detergents and analyzed in either free or liganded conformations by cryo-electron microscopy [55–58]. While these studies have provided valuable insights into Env structure, it is critical to understand the structure of the Env trimer in its native membrane environment. Changes in the unusually long (approximately 145 amino acid residues) gp41 cytoplasmic tail can affect the rate of membrane fusion mediated by Env and the antigenicity of the gp120 and gp41 ectodomains [59–62]. Thus, studies of Env in a membrane environment promise to provide a more comprehensive understanding of the structure of the functional HIV-1 Env trimer.

Nanodiscs and other lipid nanoparticles have been shown to incorporate purified integral membrane proteins into a lipid bilayer that is scaffolded by a lipid-associated protein to allow solubility in aqueous buffers [63–66]. Nanodiscs are discoidal lipid bilayer particles of 10-17-nm diameter surrounded by a membrane scaffold protein (MSP), an engineered version of Apolipoprotein A1 [63–65]. Nanodiscs have been shown to incorporate several previously refractory membrane proteins, allowing biochemical and structural characterization [67–72]. Recently, nanodiscs and saposin-lipid nanoparticles have been used to incorporate HIV-1 Env complexes, which retained antigenicity and were stable for days at low temperature [66,73]. ND resistance to shear and thermal stress may assist efforts to create practical vaccine platforms. Here, we describe approaches to optimize the incorporation of HIV-1 Envs into NDs, and characterize the antigenicity and stability of the incorporated Envs.

## Materials and methods

### HIV-1 Envs

The HIV-1_JR-FL_ Env(-) is a full-length Env with arginine residues 508 and 511 modified to serine residues, preventing furin proteolytic cleavage of the Env precursor [74–76]. The HIV-1_JR-FL_ Env(-)Δ808 is identical to the Env(-) glycoprotein except that the cytoplasmic tail has been truncated, with residues 808–856 deleted. Both Env variants have a CD5 signal sequence, and a Gly-Gly spacer followed by a (His)_6_ sequence at the carboxyl terminus. Env residues are numbered according to the HIV-1_HXBc2_ prototypic sequence, as per current convention [77,78].

### Env-expressing cells

**Fig 1:**
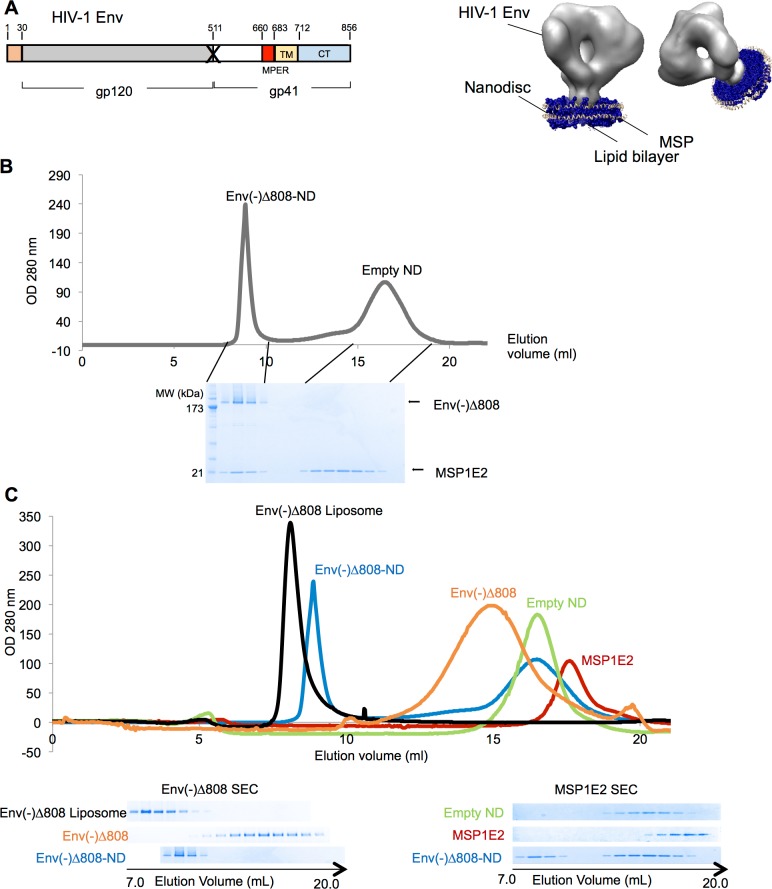
Components of HIV-1 Env-NDs. (A) A diagram of the wild-type (WT) HIV-1 Env is shown, with the signal sequence (S), gp120-gp41 cleavage site (after residue 511), and transmembrane region (residues 683–712). The HIV-1_JR-FL_ Env(-) glycoprotein is identical to the WT Env except for changes at residues 508 and 511, in the sequence for gp120-gp41 cleavage (designated with an X). The Env(-)Δ808 glycoprotein is identical to the Env(-) glycoprotein except for a deletion of the carboxyl terminus (residues 808–856). An idealized diagram of an Env-ND is shown on the right, with the HIV-1 Env trimer, membrane scaffolding protein (MSP) and lipid bilayer. The depiction of Env is based on cryoelectron tomograms from reference 50. (B) An assembly reaction with the HIV-1_JR-FL_ Env(-)Δ808 glycoprotein, MSP1E2 and brain lipids was analyzed by size-exclusion chromatography (SEC). The SEC fractions were analyzed by SDS-PAGE and Coomassie Blue staining. The peaks corresponding to the Env(-)Δ808-NDs and the empty NDs are labeled. (C) Size-exclusion chromatograms of the HIV-1_JR-FL_ Env(-)Δ808 glycoprotein assembled into NDs, Env(-)Δ808 in liposomes, Env(-)Δ808 in detergent, MSP1E2 protein and empty NDs are superimposed. In the bottom panels, the proteins in the eluted fractions were analyzed by SDS-PAGE and Coomassie Blue staining. The Env(-)Δ808 glycoprotein in the SEC fractions is shown in the left panel, and the MSP1E2 protein in the right panel.

**Fig 2:**
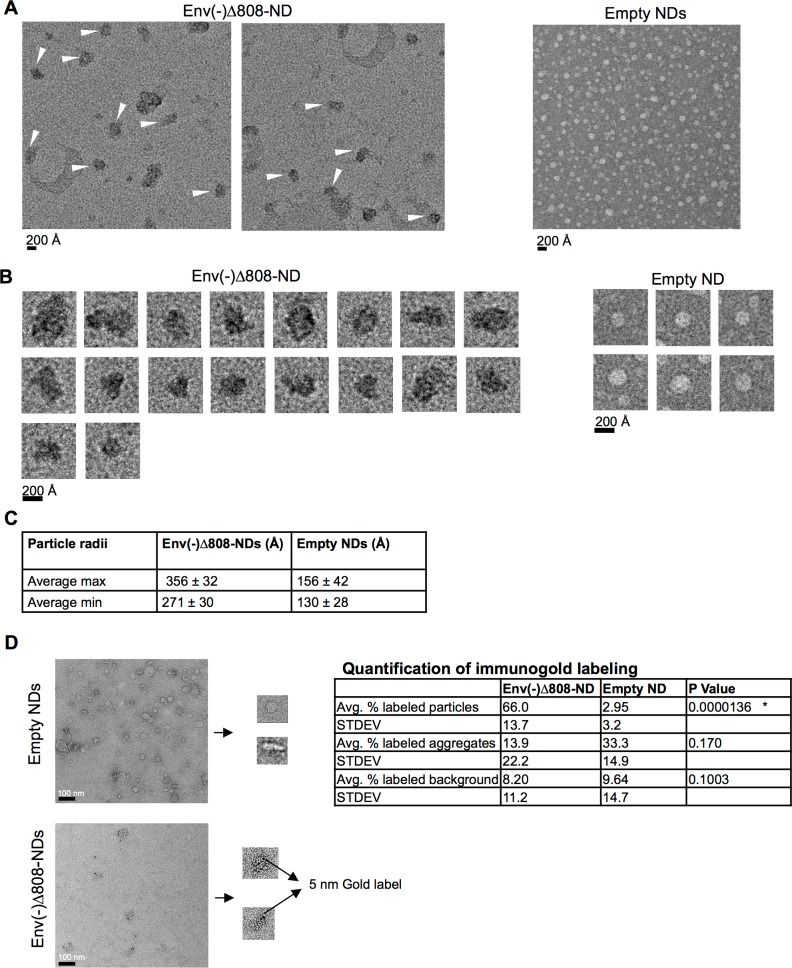
Morphology and size of Env-NDs and empty NDs. (A) Negative-stain electron micrographs of HIV-1_JR-FL_ Env(-)Δ808-NDs and empty NDs. (B) Examples of individual particles in the preparations of Env(-)Δ808-NDs and empty NDs. (C) One hundred randomly chosen Env(-)Δ808-NDs and empty NDs were measured, and both the maximum and minimum dimensions were recorded and averaged. The Env(-)Δ808-ND particles that clearly consisted of aggregated individual NDs were excluded from the analysis. (D) The Env(-)Δ808-NDs and empty NDs were incubated with the 2G12 antibody and Protein A-5 nm gold conjugate. For the empty NDs, 244 particles were examined; for the Env(-)Δ808-NDs, 44 particles were examined. The percentage of labeled particles was calculated. The gold beads associated with large aggregates and with the randomly sampled non-particulate background were also scored. Statistical significance was evaluated by using a Student’s t-test.

**Fig 3:**
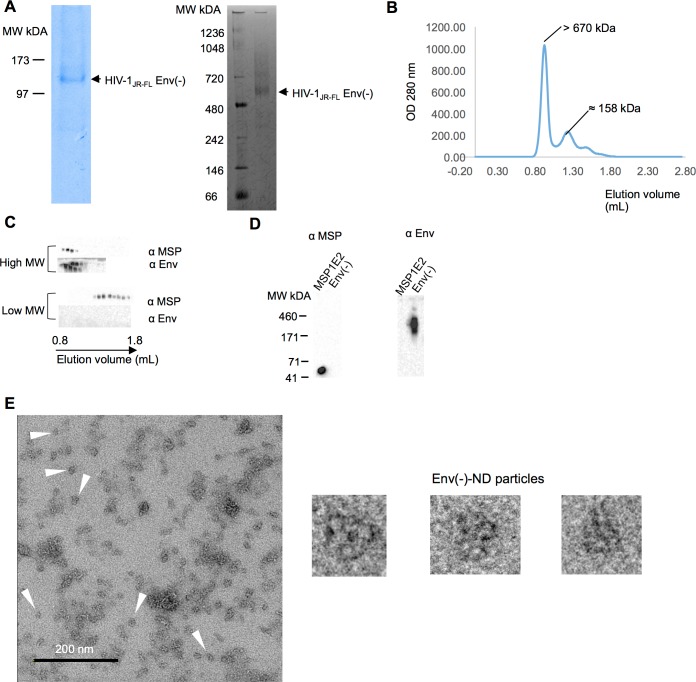
Incorporation of the full-length HIV-1_JR-FL_ Env precursor into NDs. (A) The HIV-1_JR-FL_ Env(-) glycoprotein was purified and analyzed by SDS-PAGE (left panel) and Blue Native PAGE (right panel). Both gels were stained with Coomassie Blue. (B) A size-exclusion chromatograph (SEC) of the glutaraldehyde-crosslinked HIV-1_JR-FL_ Env(-)-NDs run on a Superdex 200 column is shown. (C) The high-molecular-weight SEC fractions (upper panel) and the low-molecular-weight SEC fractions (lower panel) were Western blotted and probed with a mixture of sera from HIV-1-infected individuals to detect Env (αEnv) and with an anti-ApoA1 antibody to detect MSP1E2. An anomaly in the gel caused some of the Env bands in the high-molecular-weight SEC fractions to appear artefactually as two bands. (D)The specificities of the anti-ApoA1 antibody and the serum mixture for MSP1E2 and Env(-), respectively, were demonstrated by probing Western blots. (E) Negative-stain electron micrograph of glutaraldehyde-crosslinked HIV-1_JR-FL_ Env(-)-NDs and selected particles. The white arrows indicate individual Env(-)-NDs; some aggregates of the Env(-)-NDs are evident as well.

**Fig 4:**
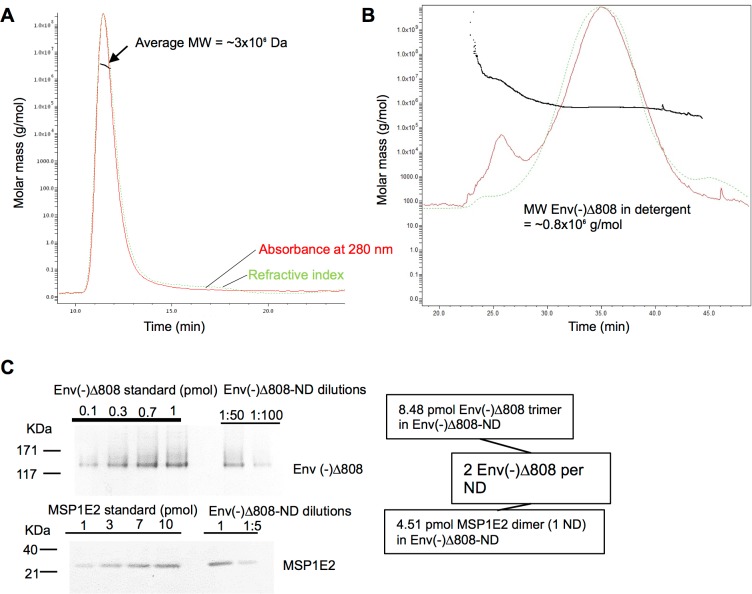
Molecular weight and composition of the Env-NDs. (A) The HIV-1_JR-FL_ Env(-)Δ808-NDs were analyzed by Multi-Angle Light Scattering (MALS). (B) MALS analysis of the purified Env(-)Δ808 glycoprotein in Cymal-6 detergent. (C) Krypton staining of SDS-polyacrylamide gels of dilutions of the Env(-)Δ808-NDs, which were compared with Krypton-stained standards (known amounts of Env(-)Δ808 glycoprotein and MSP1E2). The densitometric quantification of the bands allowed an estimation of the average Env(-)Δ808: MSP1E2 ratio in the Env-NDs.

**Fig 5:**
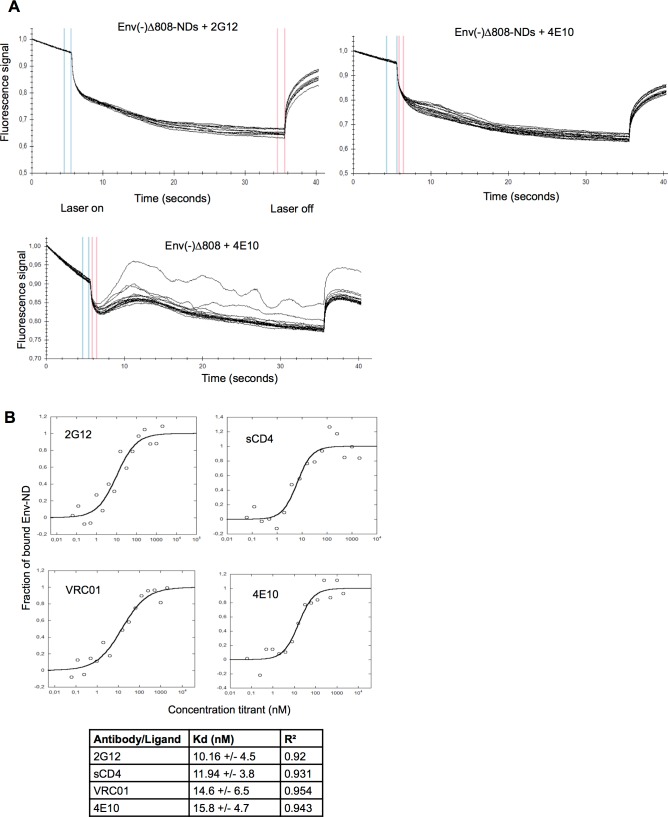
Microscale thermophoresis measurement of ligand interaction with Env-NDs. (A) Microscale thermophoresis profiles of the interaction of the 2G12 and 4E10 antibodies with HIV-1_JR-FL_ Env(-)Δ808-NDs are shown in the two upper panels. In the lower panel is shown a microscale thermophoresis profile of the 4E10 antibody with the purified Env(-)Δ808 glycoprotein in Cymal-6 detergent. Blue and red lines indicate laser activation and inactivation respectively to produce the thermal gradient. (B) Microscale thermophoresis was used to determine the saturation binding curves for the Env(-)Δ808-NDs of sCD4 and the 2G12, VRC01 and 4E10 antibodies. The calculated dissociation constants are shown beneath the curves. The binding of the 17b antibody to the Env(-)Δ808-NDs was below the limits of detection.

**Fig 6:**
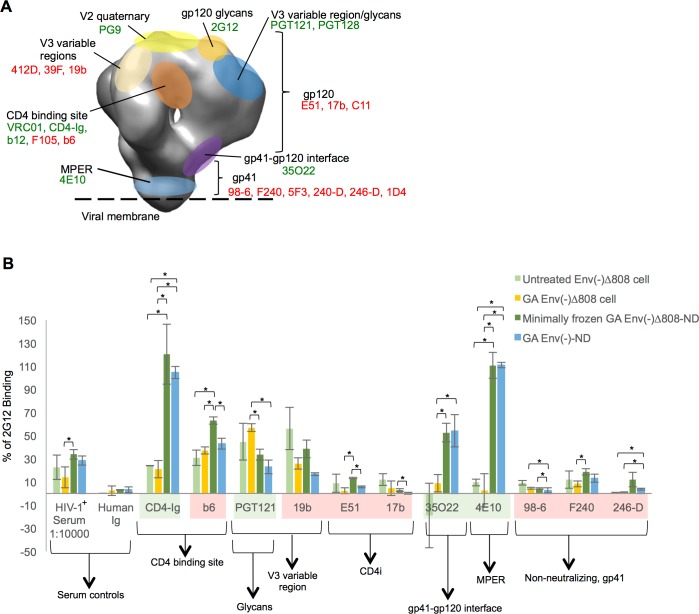
Antigenic characterization of the HIV-1_JR-FL_ Env(-)Δ808-NDs and glutaraldehyde-treated and untreated Env(-)Δ808 glycoproteins on the surface of expressing cells. (A) The epitopes on the HIV-1 Env trimer recognized by the monoclonal antibodies used in this study are shown. The HIV-1 Env structure shown in schematic is from reference 50. The potently neutralizing antibodies that are expected to bind the Env trimer efficiently are shown in green text. The weakly neutralizing antibodies that are not expected to bind the HIV-1 Env trimer efficiently are shown in red text. (B) Binding of the indicated ligands to the minimally frozen HIV-1_JR-FL_ Env(-)Δ808-NDs or the HIV-1_JR-FL_ Env(-)-NDs captured on an ELISA plate is compared with ligand binding to glutaraldehyde-treated or untreated Env(-)Δ808 glycoprotein in a cell-surface ELISA. * = p < 0.05, unpaired t-test.

**Fig 7:**
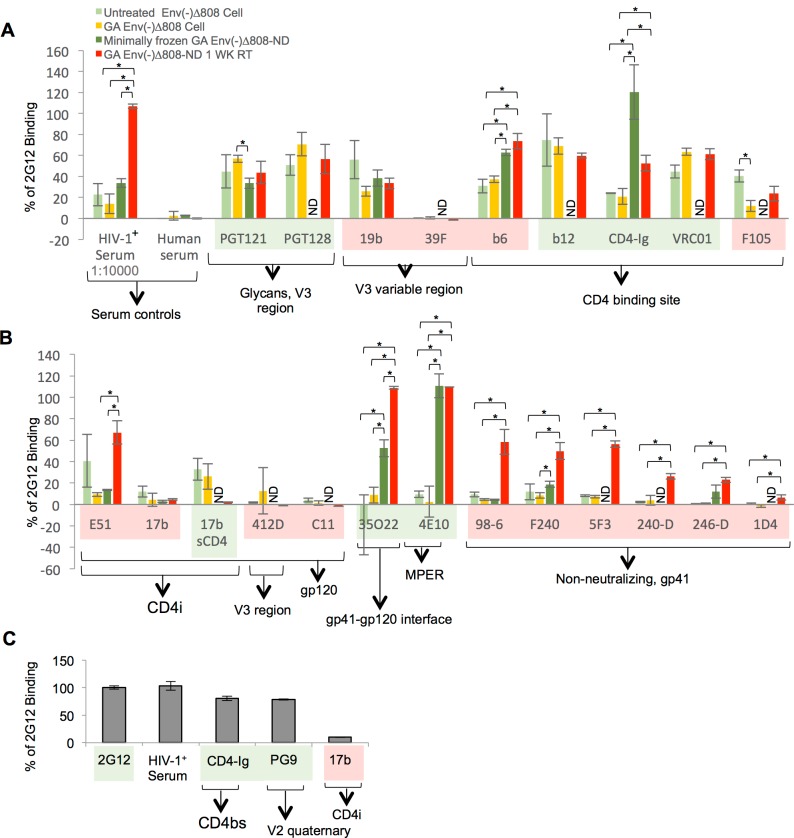
Effect of prolonged room-temperature incubation on the antigenicity of HIV-1 Env-NDs. (A,B) Binding of the indicated ligands to the HIV-1_JR-FL_ Env(-)Δ808-NDs that had been incubated at room temperature for a week is compared to that of the same ligands to glutaraldehyde-treated and untreated HIV-1_JR-FL_ Env(-)Δ808 glycoprotein on the surface of expressing cells. Shading of the ligands is described in the legend to Fig 6. (**C**) The binding of the indicated ligands to the HIV-1_JR-FL_ Env(-)Δ808 E168K-NDs that had been incubated at room temperature for one week is shown. The E168K change allows the HIV-1_JR-FL_ Env to be recognized by the V2 quaternary-dependent antibody PG9 (101). * = p < 0.05, unpaired t-test. ND–Not determined.

**Fig 8:**
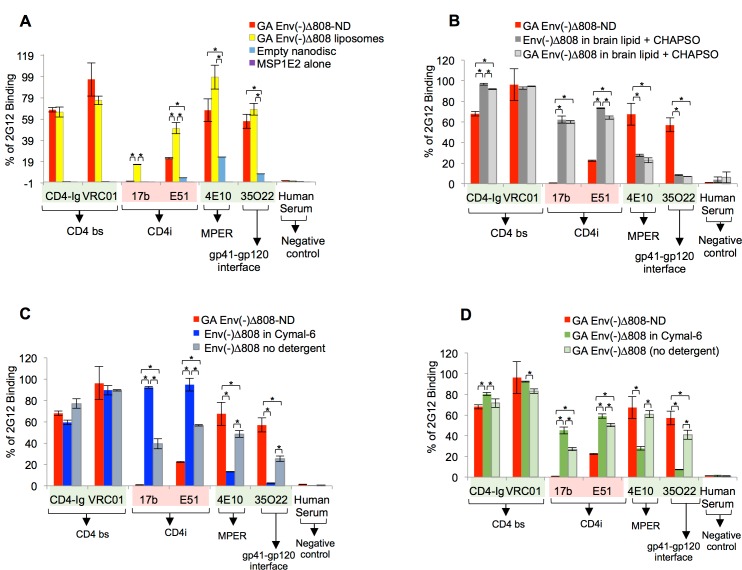
Effect of a one-week room temperature incubation of HIV-1 Env in NDs, liposomes, detergent and in detergent-free conditions. (A-D) The binding of the indicated ligands to the HIV-1_JR-FL_ Env(-)Δ808 glycoprotein in NDs (glutaraldehyde-crosslinked), liposomes (crosslinked), in brain lipids/CHAPSO, in Cymal-6, or in detergent-free conditions was measured by ELISA. The ligands are highlighted as described in the Fig 6 legend. * = p < 0.05, unpaired t-test.

**Fig 9:**
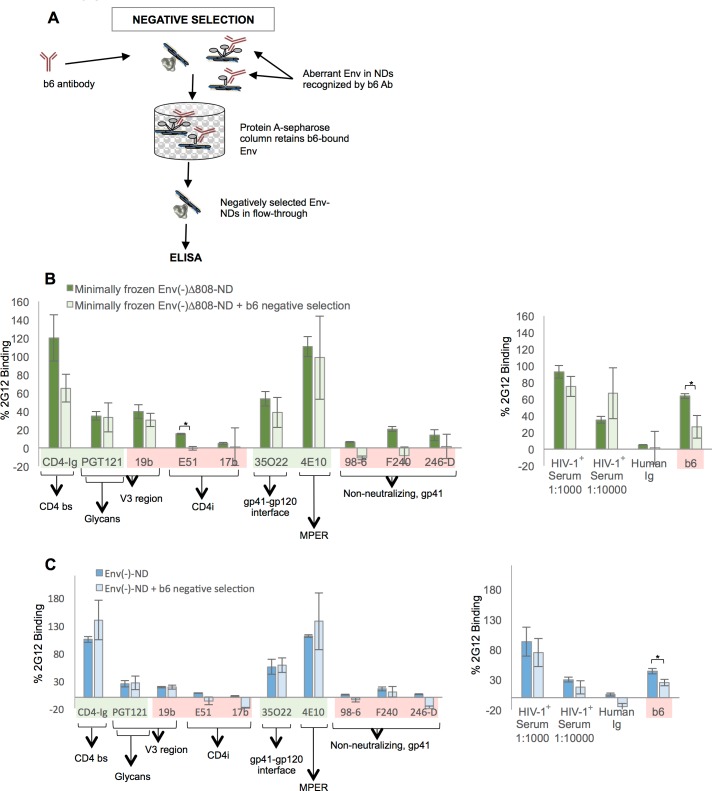
Negative selection of Env-NDs with the weak neutralizing antibody b6. (A) Schematic diagram of negative selection of Env-NDs with the weakly neutralizing b6 antibody. (B,C) The recognition of the HIV-1_JR-FL_ Env(-)Δ808-NDs and Env(-)-NDs captured on an ELISA plate by the indicated ligands is shown. The ligands are highlighted as described in the legend to Fig 6. * = p < 0.05, unpaired t-test.

Chinese hamster ovary (CHO) cells inducibly expressing the HIV-1_JR-FL_ Env(-)Δ808 and Env(-) glycoproteins were established as described in Go *et al*., 2015 [79]. CHO cells were grown in 1:1 HyClone CDM4CHO and CDM4HEK293 medium (Fisher), supplemented with 1X L-glutamine, 1X Penicillin Streptomycin L-glutamine and 0.05% anti-clumping agent (Life Technologies). Cells were cultured in 2-L vented roller bottles (Fisher) rotating at 10 rpm, and passaged over five times before induction with 1–1.5 μg/mL doxycycline (Fisher) at a cell density greater than 5 million cells/mL. Between 15 and 24 hours after induction, CHO cells were harvested and either used directly for Env purification as described [57,79] or frozen for later use. Plasma membranes were extracted from CHO cells using two protocols: Protocol 1) Frozen CHO cells were homogenized in solubilization buffer (250 mM sucrose, 20 mM HEPES, pH 7.4 with protease inhibitors (Gilead Sciences)) with a glass Dounce homogenizer. The homogenate was then centrifuged at 1000 x g for 10 minutes at 4°C, and the supernatant was passed over a glass wool filter, then ultra-centrifuged (100,000 x g for 30 min at 4°C) to pellet cell membranes, which were dried briefly at room temperature before plunge freezing or solubilization. Protocol 2) The CHO cell homogenate was centrifuged at 1000 *x* g for 10 minutes at 4°C to pellet cell debris. The supernatant was then centrifuged at 10,000 x g for 10 minutes at 4°C to pellet mitochondria. Plasma membranes were then pelleted via ultracentrifugation at 100,000 x g for 30 minutes at 4°C, re-suspended in 20 mM Tris-HCl, pH 7.4 (Sigma), 300 mM NaCl (Sigma), 100 mM (NH_4_)_2_SO_4_ (Hampton Research), 0.02% NaN_3_ (Sigma), then pelleted again at 100,000 x g for 45 minutes at 4°C. The Envs used in each experiment were prepared according to the indicated protocol: Fig 1 (Protocol 1), Fig 2A–2C (Protocol 1), Fig 2D (Protocol 2), Fig 3 (Protocol 2), Figs 4 and 5 (Protocol 1), Fig 6 (Protocol 2), Figs 7 and 8 (Protocol 1), Fig 9 (Protocol 2) and S1–S3 Figs (Protocol 1).

### Purification of membrane Env

Cell membrane Env was solubilized using a Dounce homogenizer on ice in 20 mM Tris HCl, pH 7.4, 300 mM NaCl, 100 mM (NH_4_)_2_SO_4_, 20 mM imidazole (MP Biomedical), protease inhibitors, and detergent (7.2 mM Cymal-5 and 0.25% Tween-80 for optimized Env purification). We evaluated different detergents in place of Cymal-5/Tween-80: 0.5% Brij-98, 7.2 mM Cymal-5, 0.5% Triton X-100, 1 mM NP40, 6 mM CHAPS, 0.25% Tween-80, or 8 mM CHAPSO (Anatrace and Sigma). Solubilized membranes containing Env were filtered with a 0.22-μm filter, then passed over a Hi-Trap or Talon Ni-NTA column equilibrated in solubilization buffer. After washing with solubilization buffer, Env was eluted in 20 mM Tris HCl, pH 7.4, 300 mM NaCl, 100 mM (NH_4_)_2_SO4, 500 mM imidazole, 0.012 mM Tween-80 or with an imidazole gradient as needed to improve Env purity. Purified Env was analyzed by size-exclusion chromatography (SEC) in 20 mM Tris HCl, pH 7.4 and 300 mM NaCl and detergent at the critical micelle concentration (CMC) on a Yarra 4000 column on an Akta Micro purifier, followed by semi-dry Western blotting of SEC fractions probed with anti-gp120 primary antibody (Abcam) or a mixture of sera from HIV-1-infected individuals. Protein A-Horseradish Peroxidase (Invitrogen) and West Femto Luminol reagent (Thermo Scientific) were used for signal detection. The purified HIV-1_JR-FL_ full-length Env(-) was analyzed on a Novex Native PAGE 4–16% Bis-Tris gel (ThermoFisher), followed by Coomassie Blue staining.

### Env immunoprecipitation assays

Oligomeric Env fractions from SEC were diluted to approximately 0.01 mg/mL and incubated with approximately 1 microgram of each anti-Env antibody for 1–2 hours on ice. Aliquots of 15–20 μl of Protein A-Sepharose beads (Life Technologies or Sigma) were blocked in blocking buffer (20 mM Tris HCl/HEPES, pH 7.4, 300 mM NaCl, 2–10% BSA), then equilibrated in 20 mM Tris HCl/HEPES, pH 7.4, 300 mM NaCl, and detergent at the CMC. Beads and Env-Ab complexes were incubated briefly on ice, then vortexed and spun down. Supernatant was withdrawn, and both beads and supernatant were analyzed by SDS-PAGE and Western blot analysis.

### MSP1E2 purification

Plasmids for membrane scaffold protein (MSP) expression were a kind gift of Dr. Stephen Sligar (University of Illinois) through the Addgene program. MSP constructs were purified as described [63]. Briefly, the MSP-containing cell lysate was pelleted, and supernatant was filtered with a 0.45-μm filter before passage over a Ni-NTA column equilibrated with 1X PBS and 1% Triton X-100. The column was washed with lysis buffer, then with repeated washes of 40 mM Tris HCl, 300 mM NaCl, 1% Triton X-100, followed by washes with 1% 50 mM cholate (Sigma) and 20 mM imidazole pH 7.4, and finally 50 mM imidazole, with additional washes as necessary to clarify the resin. MSP was eluted in fractions of 40 mM Tris/HCl, 300 mM NaCl, 500 mM imidazole, pH 8.0, or an imidazole gradient as needed to achieve the desired purity of MSP. Fractions were checked by SDS-PAGE electrophoresis and Coomassie Blue staining (Invitrogen), and fractions containing MSP were collected and dialyzed overnight at 4°C in 20 mM Tris HCl, pH 7.4, 100 mM NaCl, 0.5 mM EDTA. Dialyzed MSP was collected and concentrated, then further purified by SEC as needed before plunge-freezing and storing at -80°C.

### Lipid and detergent screening

Porcine brain, bovine liver, and bovine heart total lipid extracts, DMPC, and POPC from Avanti Polar Lipids were dried under nitrogen gas (Airgas) and then placed in a vacuum for 1 hour to overnight. The lipids were solubilized in 20 mM Tris HCl, pH 8 and 300 mM NaCl and detergent (CHAPSO, CHAPS, NP40, Brij-98, Cymal-5, Tween-80 or Triton X-100) at a concentration approximately twice the lipid concentration. Lipids were heated, vortexed, diluted, and sonicated as necessary to achieve complete solubilization. Detergent removal was achieved using 30 BioBeads SM-2 Adsorbent (BioRad) (freshly washed with methanol and then three times with water and dried) per 20 μL reaction (a “standard reaction”) either overnight at 4°C or 3 hrs rotating at room temperature (or 4°C for POPC). Reactions were filtered, then applied to a Yarra 4000 SEC column (Phenomenex) in 20 mM Tris HCl, pH 8 and 300 mM NaCl without detergent to screen for the formation of high-molecular-weight species.

### Env-ND assembly

The above-determined conditions allowing successful liposome formation were used in combination with 5 μL of concentrated Env (2–5 mg/mL) to assemble Env-NDs. In a “standard reaction,” detergent was removed with 30 BioBeads per 20 μL reaction for 3 hours at room temperature, rotating. Reactions were filtered with a 0.22-μm filter, then passed over a Yarra 4000 SEC column to identify conditions allowing large complex formation. SEC fractions were Western blotted and probed with an anti-gp120 antibody.

Based on the results of the screening assays described above, the following protocol was used to prepare Env-NDs. Brain lipids (5 mg), dried under nitrogen and placed in a vacuum for 1 hour, were solubilized in 410 μL of 80 mM CHAPSO reconstitution buffer (20 mM Tris HCl, pH 8 and 300 mM NaCl) using constant heating at 50°C alternated with six rounds of 5-minute incubations at 100°C, followed by one hour of sonication at 50°C. Lipids were pelleted, filtered through a 0.22-μm filter, and heated until clear, then allowed to cool to room temperature. Then, 10 μL of the cooled lipid preparation was incubated with 5 μL of Env (concentrated between 3–7 mg/mL by Nanodrop, measured by absorbance at 280 nm (extinction coefficient of 1.9 (mg/mL)^-1^ cm^-1^), and 5 μL of MSP (concentrated to 9–20 mg/mL), in a standard 20-μL reaction. As part of the screening, we evaluated MSP2, MSP1E2, MSP2E2, MSP1E1, MSP1D1, MSP1E3 and MSP1E3D1. Reactions were incubated with 30 BioBeads to remove detergent over 4 hours at room temperature, rotating. Reactions were then filtered and analyzed by SEC and Western blotting as described above. For Env-ND assembly with lipopolysaccharide (LPS) and lipoarabinomannan from M. smegmatis (LAM-MS) [80,81], LPS (Sigma) was combined with assembly lipids at a final concentration of 1% of total lipids, and LAM-MS (Invivogen) was used at 5 μg/mL final concentration, as recommended by suppliers.

### Optimized protocol for Env-ND assembly

Env-NDs were assembled as described above, with 5 μL of concentrated MSP1E2 (9–20 mg/ml) per total 20 μL standard reaction. After assembly, Env-NDs were passed through a Yarra 4000, Superdex 200, or Superose 6 10/300 GL column (GE Life Sciences) on an Akta purifier, depending on scale, at room temperature. Eluted fractions were immediately crosslinked at room temperature with 5 mM glutaraldehyde (Sigma), and reactions were either plunge frozen and stored in liquid nitrogen or -80°C, or stored at room temperature for short-term storage.

### Negative-stain electron microscopy analysis

Env-NDs were analyzed by negative staining with a Tecnai F20 transmission electron microscope (TEM) with a 4K x 4K ultra-scan CCD (Gatan) at the Center for Nanoscale Systems, Cambridge, MA, at 50k magnification. Env-NDs were applied for 1 minute to 200-Cu carbon mesh formvar grids (Electron Microscopy Sciences) glow-discharged at 25 mA for 30 seconds. Grids were rinsed in gel filtration buffer, then stained with 2% uranyl acetate for 5 minutes. Cryogrids were prepared as described [57], using an FEI Vitrobot cryoplunger. Empty nanodiscs were created and characterized by TEM as described for Env-NDs, using buffer in place of Env in the nanodisc assembly reaction. Negative-stain TEM micrographs of empty NDs were taken at 80k magnification, 120 kv and -2 μm defocus. Measurements of Env-NDs and empty NDs were done in Fiji and EMAN2.1 [82,83].

### Env-ND microscale thermophoresis

Ligand binding to purified Env-NDs was analyzed by microscale thermophoresis [84] on a Monolith NT.115 (Nanotemper Technologies). The glutaraldehyde-crosslinked HIV-1_JR-FL_ Env (-)Δ808-ND sample was fluorescent, precluding the need for labeling with an additional fluorophore. The binding of the 2G12 antibody to the Env(-)Δ808-NDs was tested using antibody concentrations ranging from 0.061 nM to 2 μM, in 20 mM Tris-HCl pH 7.5, 300 mM NaCl. The glutaraldehyde-crosslinked Env(-)Δ808-ND sample was added to a final constant concentration of 20 nM, followed by a 1-hr incubation at room temperature. The Env(-)Δ808-ND concentration was chosen such that the observed fluorescence was approximately 1,000 U at 50% LED power. The samples were loaded into Monolith capillaries and were measured by standard protocols of temperature shifts using a Monolith NT.115 (NanoTemper) as described by the manufacturer. The changes in the fluorescent thermophoresis signal were plotted against the concentration of the serially diluted antibody, and Kd values were determined using the NanoTemper analysis software.

### Env-ND Multi-Angle Light Scattering (MALS) analysis

Purified Env-NDs were passed through a Wyatt DAWN Heleos II EOS 18-angle laser photometer coupled to a Wyatt Optilab TrEX differential refractive index detector. Data were analyzed using Astra 6 software (Wyatt Technology Corp).

### Env-ND immunogold labeling

Gold Protein A 5 nM and 2G12 were incubated at room temperature at a 2:1 molar ratio. Complexes were then incubated for 10 minutes at room temperature with buffer, empty NDs or crosslinked, SEC-purified HIV-1_JR-FL_ Env(-)Δ808-NDs. Reactions were run on a Superose 6 PC 3.2/30 column with 20 mM Tris-HCl, pH 7.5, 300 mM NaCl. Fractions corresponding to ND peaks were analyzed by negative-stain TEM as described above.

### Env-ND and empty ND ELISA protocol

Env-ND and empty ND reactions (typically 300–400 μL reaction volume) were diluted in 20 mM HEPES, pH 7.4 and 300 mM NaCl to an appropriate volume, then dispensed into the wells of a white nickel-coated 96-well plate (Pierce) and incubated overnight. The Env-ND and empty ND samples were then withdrawn from the wells, and plates were washed three times with washing buffer (20 mM Tris HCl, pH 7.4, 150 mM NaCl, 20 mM imidazole), then blocked with blocking buffer (10% BSA (Sigma) in washing buffer) for 0.5–2 hours. Wells were washed three times with washing buffer, then incubated for one hour with antibody diluted to 10 μg/mL in blocking buffer. Antibodies 98–6, F240, 5F3, 240-D, 246-D, 39F, E51 (stock 2), PGT121, PGT128, F105, 4E10, 35O22, b6, b12, VRC01, and 412D were obtained from the NIH AIDS Reagent Program. 2G12 was purchased from Polymun. 1D4 was purchased from Abcam. Antibodies 17b, 19b, PG9, 35O22, E51 (stock 1), and C11 were produced from transfected HEK293F cells, with purification of the supernatant by Protein A. Antibody concentration was measured with a BCA kit (Thermo Fisher), and antibodies were aliquoted and stored at -80°C. For ELISA, after antibody binding, wells were washed three times with washing buffer, then incubated with Protein A-horseradish peroxidase (Protein A-HRP) 1:4000 in blocking buffer for 1–12 hr. Wells were washed three times with washing buffer and TBS. Wells were then probed with West Luminol Reagent Lightning ECL (Perkin Elmer), and luminescence measured with a Mithras LB 940 luminometer (Berthold Technologies). For freeze-thaw cycles, Env-NDs on ELISA plates were stored at -20°C for seven days, then brought to room temperature. Antibodies were stripped from the wells by incubation with 3 M magnesium chloride (Life Technologies) at room temperature for 30 minutes, and then Env-ND plates were washed and blocked for subsequent ELISA. For urea denaturation, Env-ND plates were incubated with 8 M urea (Sigma) for thirty minutes, washed extensively with washing buffer, then blocked for ELISA. The background signal from the empty ND ELISA was subtracted from the signals from all Env-ND ELISAs and the resulting values were then normalized to 2G12 binding.

### Immunoprecipitation of Env-NDs

Env-NDs were diluted to approximately 0.01 mg/mL and incubated with approximately 1 microgram of each antibody for 1–2 hours at room temperature. Aliquots of 15–20 μl of Protein A-Sepharose beads (Life Technologies or Sigma) were blocked in blocking buffer (20 mM Tris HCl/HEPES, pH 7.4, 300 mM NaCl, 2–10% BSA), then equilibrated in 20 mM Tris HCl/HEPES, pH 7.4, 300 mM NaCl, and detergent at the CMC. Beads and Env-ND:Ab complexes were incubated briefly on ice, then vortexed and spun down. Supernatant was withdrawn, and both beads and supernatant were prepared for SDS-PAGE and Western blot analysis.

### Cell-based ELISA

CHO cells expressing the HIV-1_JR-FL_ Env(-)Δ808 glycoprotein and initially grown in suspension were adapted for adherent growth in DMEM/F12 growth medium supplemented with 10% FBS, 1X Pen Strep glutamine, and 1:500 primocin (Invivogen). After 4–5 passages, confluent CHO cells were seeded 1:15 in a white 96-well plate. After reaching confluency, cells were induced with 1–1.5 μg/mL doxycycline in fresh growth medium. After 15–20 hours of induction, medium was removed and cells were washed twice in HEPES washing buffer (20 mM HEPES, 1.8 mM CaCl_2_ (Life Technologies), 1 mM MgCl_2_ (Life Technologies), 140 mM NaCl). Buffer was removed and cells were incubated with 5 mM glutaraldehyde in HEPES washing buffer for 25 minutes. Untreated cells were left in HEPES washing buffer. Cells were washed with 50 mM Tris HCl, pH 7.4, 1.8 mM CaCl_2_, 1 mM MgCl_2_ and 140 mM NaCl to quench the crosslinking reaction. A cell-based ELISA was then performed as described previously [85]. Cells were probed with HRP enzyme oxidizing and luminol reagents (Perkin Elmer Life Sciences) supplemented with 150 mM NaCl. Luminescence was measured with a Mithras LB 940 luminometer (Berthold Technologies). The results of the cell-based ELISAs were averaged from three separate experiments. The background ELISA signal from non-induced/crosslinked CHO cells was subtracted from the values obtained from the induced/crosslinked cells and the resulting values were then normalized to 2G12 binding.

### Env-ND negative selection

Minimally frozen Env-NDs (600-μL reaction volume) were prepared with HIV-1_JR-FL_ Env(-)Δ808 purified from CHO cells, with no freezing steps before a final plunge-freeze and storage in liquid nitrogen, using the procedures described above for Env purification. After Env-ND assembly and GA crosslinking, the HIV-1_JR-FL_ Env(-)Δ808-NDs were plunge frozen again in liquid nitrogen and stored for 1 week. A 200-μL reaction was thawed and used for negative selection. A 60-μL volume of crosslinked HIV-1_JR-FL_ Env(-)-NDs was prepared from the purified Env(-) glycoprotein, assembled using the Env-ND assembly protocol, and stored at -80°C. The b6 antibody was incubated with thawed Env(-) and Env(-)Δ808 NDs at a final concentration of 0.15 mg/mL. Reactions were diluted 100-fold in 20 mM HEPES, pH 7.4, 300 mM NaCl, and applied to a 30-μL bed of Protein A-Sepharose beads (extensively washed in water, buffer, and blocked with 10% BSA in buffer). Control reactions of Env-NDs without added antibody were incubated at room temperature, and applied to Protein A-beads as well. Flowthrough was reapplied to the Protein A-column, and the final flow-through was diluted appropriately, and applied to a nickel-coated white 96-well plate at a volume of 50 μL per well. ELISA was performed as described above for Env-NDs. The background binding from the average signal for blocking buffer was subtracted from the signals for the Env(-) and minimally frozen Env(-)Δ808-NDs, and the resulting values were then normalized to 2G12 binding. The background binding to empty NDs was subtracted from the ELISA results for Env(-)Δ808-ND incubated at room temperature for one week, and the resulting values were then normalized to 2G12 binding.

## Results

### Screening Envs, detergents and lipids to optimize HIV-1 Env-ND assembly

To optimize the assembly of HIV-1 Env-NDs, we tested several variables that potentially influence this process and the resulting quality of the incorporated Env. The gp41 cytoplasmic tail has been suggested to influence the conformation of the HIV-1 Env ectodomain [59–62]. Therefore, in these studies, we used either full-length HIV-1 Env or a truncated Env (Env(-)Δ808) that retains all of the gp41 cytoplasmic tail residues that significantly influence Env function or antigenicity [59–62] (Fig 1A). The Envs were derived from HIV-1_JR-FL_, a primary, neutralization-resistant strain. To increase the stability of Env, these studies were carried out using the uncleaved Env precursors, Env(-) and Env(-)Δ808 glycoproteins, produced in Chinese hamster ovary (CHO) cells [79].

Cell membranes were prepared from the CHO cells expressing the Env(-)Δ808 glycoprotein. We screened detergents for the ability to extract Env(-)Δ808 from membranes while preserving the Env oligomeric state and important Env epitopes (S1 Fig). Several detergents (Brij 98, Triton X-100, Tween 80, Nonidet P40, CHAPS, CHAPSO and Cymal-5) met these requirements. These detergents were then used to solubilize brain lipid extracts, which are similar in composition to the HIV-1 lipidome [86]. When purified HIV-1_JR-FL_ Env(-)Δ808 trimers and brain lipids in CHAPSO were mixed with the membrane scaffolding protein MSP1E2 and the detergent concentration was subsequently reduced by incubation with hydrophobic beads, complexes at a size expected for Env-NDs were observed by size-exclusion chromatography (Fig 1B). The Env(-)Δ808 glycoprotein and MSP1E2 protein represented the major protein components of these complexes. The Env(-)Δ808-ND complexes eluted from a size-exclusion column earlier than the unassembled MSP1E2 protein, the purified Env(-)Δ808 glycoprotein alone, and empty nanodiscs without

Env(-)Δ808 (Fig 1C). Importantly, essentially all of the Env(-)Δ808 glycoprotein detected in the Env-ND reaction was present in the high-molecular-weight fraction that also contained the MSP1E2 protein. Env(-)Δ808 liposomes without the MSP1E2 protein eluted earlier than Env-NDs from the size-exclusion column (Fig 1C). Size-exclusion chromatography was used to prepare Env-NDs for subsequent analysis, allowing their separation from unassembled MSP1E2 or empty NDs. Upon storage at 4°C, the Env-NDs aggregated, a process that could be prevented by crosslinking the nanodiscs with glutaraldehyde.

### Env-ND morphology and size

The Env-NDs with the HIV-1_JR-FL_ Env(-)Δ808 glycoprotein exhibited discrete particles ranging from 271–356 Angstroms in diameter, about twice the size of the empty nanodiscs (130–156 Angstroms in diameter) (Fig 2A–2C). The Env(-)Δ808-NDs were also morphologically distinct from empty nanodiscs. The Env-NDs exhibited specific staining after incubation with the 2G12 anti-gp120 antibody and Protein A conjugated to 5-nm gold beads (Fig 2D).

The full-length HIV-1_JR-FL_ Env(-) glycoprotein was purified (Fig 3A) and used to prepare Env-NDs. The presence of both the Env(-) glycoprotein and the MSP1E2 protein in the Env-NDs was documented (Fig 3B–3D). The size and morphology of the Env(-)-NDs was similar to that of the Env(-)Δ808-NDs (Fig 3E). Thus, the full-length HIV-1 Env precursor can be incorporated into NDs.

Multi-Angle Light Scattering [87,88] indicated that the Env(-)Δ808 NDs ranged in size from 1500 to 3500 kDa (Fig 4A). Multi-Angle Light Scattering analysis of the HIV-1_JR-FL_ Env(-)Δ808 glycoprotein in detergent suggested an estimated molecular weight of approximately 800 kD (Fig 4B). This indicated that multiple Env(-)Δ808 glycoproteins might be contained in each Env-ND. We calculated the average Env: MSP1E2 ratio in the Env(-)Δ808-NDs using krypton staining and densitometric quantification of gels, and comparison with protein standards (Fig 4C). These studies suggested that, on average, two Env complexes are incorporated into each nanodisc.

### Ligand binding to Env-ND

The binding of selected antibodies against gp120 and gp41 to the Env(-)Δ808-NDs was measured by microscale thermophoresis. Microscale thermophoresis measures the movement of molecules along a thermal gradient upon ligand binding [84]. We measured the binding of soluble CD4 (sCD4), three anti-gp120 antibodies (2G12, VRC01 and 17b) and an anti-gp41 antibody (4E10) to the Env(-)Δ808-NDs. The smoothness of the thermoscan profile resulting from the binding of the 2G12 and 4E10 antibodies to the Env(-)Δ808-NDs indicates that the Env-NDs are relatively homogeneous (Fig 5A). This contrasts with the thermoscan profile of the Env(-)Δ808 glycoprotein and 4E10 antibody in detergent solution, which suggests that some heterogeneity exists in the solubilized Env. Incorporation into NDs may allow Env to maintain greater homogeneity. As a side note, Env-NDs were sufficiently fluorescent to scan without the addition of fluorescently tagged ligand, a property that was found to result from glutaraldehyde crosslinking [89–91].

The thermophoresis profiles allowed the calculation of binding constants for Env ligands (Fig 5B). The conformation-dependent epitopes recognized by the 2G12, VRC01 and 4E10 neutralizing antibodies, as well as the CD4 binding site, are intact on the Env(-)Δ808-NDs, based on the high affinity of the observed binding. The gp120 epitope for the 17b antibody, which is masked on the unliganded HIV-1 Env trimer and is exposed after CD4 binding [92], was not available for binding on the Env(-)Δ808-NDs (Fig 5B legend).

We used an ELISA to evaluate the conformation of the full-length Env(-) and Env(-)Δ808 glycoproteins in the NDs. The binding of a panel of neutralizing and non-neutralizing antibodies to the Env-NDs was compared with that to Env on the surface of expressing cells or in other formulations (proteoliposomes, detergent, without detergent after crosslinking). In general, potent neutralizing antibodies bound the mature HIV-1 Env trimer more efficiently than weakly neutralizing antibodies or non-neutralizing antibodies [36–38,93–95]. The antibodies used in this study and their Env epitopes are summarized in Fig 6A.

We compared the antigenicity of the HIV-1_JR-FL_ Env(-) and Env(-)Δ808 glycoproteins in NDs, which had been prepared using glutaraldehyde treatment, with that of the HIV-1_JR-FL_ Env(-)Δ808 glycoprotein expressed on untreated or glutaraldehyde-treated CHO cells. As has been previously seen [35,85,96], the neutralizing antibody 2G12 exhibited a high level of recognition of the Envs in all contexts, and the binding of the other antibodies was thus normalized to that of 2G12 to control for the overall amount of Env in the different assays (Fig 6B). Glutaraldehyde treatment exerted minimal effects on the recognition of the Env(-)Δ808 glycoprotein on the CHO cell surface (Fig 6B); some decrease in Env(-)Δ808 recognition by non-neutralizing antibodies was associated with glutaraldehyde crosslinking, as previously reported [97,98]. Relative to the cell-surface Env(-)Δ808 glycoprotein, both the Env(-)Δ808 glycoprotein and the full-length Env(-)Δ808 glycoprotein in NDs exhibited significantly better binding of CD4-Ig and the 35O22 and 4E10 antibodies. The 35O22 antibody recognizes the gp120:gp41 interface [99] and the 4E10 antibody recognizes the membrane-proximal external region of gp41 [100]. The exposure of the Env epitopes for other antibodies in the Env-NDs was similar to that seen in the cell-surface Env(-)Δ808 glycoprotein. With the exception of the gp120 V3 epitope recognized by the 19b antibody and the CD4-binding site epitope recognized by the weakly neutralizing b6 antibody, the epitopes for weak or non-neutralizing antibodies were not well exposed on the Env-NDs. Thus, the antigenic conformation of the Envs in NDs generally resembles that of the same Envs on the expressing cell surface, with the exception of better recognition by a soluble form of the CD4 receptor and two neutralizing antibodies, 35O22 and 4E10.

Although the recognition of the Env(-)-NDs and Env(-)Δ808-NDs by the panel of Env ligands was similar, the full-length Env(-)-NDs were recognized less efficiently by several weak or non-neutralizing antibodies (Fig 6B). These included b6 (CD4-binding site), 19b (gp120 V3 region), E51 and 17b (CD4-induced gp120 epitopes) and 98–6 (a non-neutralizing gp41 epitope). Thus, the full-length Env-NDs may be slightly more efficient in masking the epitopes for these poorly neutralizing antibodies.

### Preservation of Env antigenicity over time in Env-NDs

We evaluated the stability of the Env conformation in NDs by incubating the Env(-)Δ808-NDs at room temperature for one week and examining recognition by the panel of Env ligands. After the one-week incubation at room temperature, the

Env(-)Δ808-NDs retained the epitopes for all of the antibodies that recognized the Env-NDs prior to the incubation (Fig 7A and 7B). We also tested an HIV-1_JR-FL_ Env(-)Δ808 E168K variant, which retains the gp120 V2 region quaternary epitope recognized by the PG9 antibody [101]. After a one-week room temperature incubation, the PG9 antibody efficiently bound the Env(-)Δ808 E168K-NDs (Fig 7C). Thus, the overall architecture of the Env(-)Δ808 glycoprotein appears to be preserved after a week-long incubation of the NDs at room temperature.

Room temperature incubation for one week resulted in increases in exposure of some epitopes recognized by weak or non-neutralizing antibodies: b6 (CD4-binding site of gp120), E51 (CD4-induced epitope of gp120), and several antibodies against the gp41 ectodomain (98–6, F240, 5F3, 240-D, 246-D and 1D4) [102,103] (Fig 7A and 7B). Room temperature incubation also resulted in increased Env(-)Δ808 recognition by the serum from an HIV-1-infected individual (Fig 7A), consistent with the abundance of poorly neutralizing antibodies directed against the CD4-induced gp120 epitopes and gp41 ectodomain epitopes in most HIV-1-infected individuals [104–109]. These results indicate that some masking of non-neutralizing Env epitopes is compromised in the Env-NDs after one week at room temperature. Nonetheless, the exposure of the gp120 CD4-induced epitope recognized by the 17b antibody was not available for binding after a one-week room temperature incubation of the Env-NDs (Fig 7B).

We also compared the effects of a one-week incubation at room temperature on the antigenic profile of the Env(-)Δ808 glycoprotein in different contexts: Env-NDs, Env in liposomes, Env in Cymal-6 detergent, Env in the absence of detergents, and Env in solubilized lipids (Fig 8A–8D). The 17b and E51 antibodies against CD4-induced gp120 epitopes recognized the Env(-)Δ808 glycoprotein significantly better in contexts other than Env-NDs. The Env-NDs preserved the epitopes for the VRC01, 4E10 and 35O22 neutralizing antibodies at least as well as the other contexts. These results indicate that the ND environment preserves Env architecture better than the other contexts.

Additional environmental stress was applied to the HIV-1_JR-FL_ Env(-)Δ808-NDs that had been incubated for one week at room temperature. One freeze-thaw minimally affected epitope exposure on the Env-NDs (S2 Fig). Two cycles of freeze-thawing or treatment with urea resulted in significant changes in multiple Env epitopes (S2 Fig), suggesting that the global architecture of the Env(-)Δ808 glycoprotein in NDs can be altered by strong denaturants.

### Negative selection of Env-NDs with an antibody

The counter-selection of Env molecules in a population with non-neutralizing or weakly neutralizing antibodies has been employed to attempt to produce HIV-1 Env immunogens that better mask these epitopes from the host immune system [110–114]. We evaluated whether the Env(-)Δ808-NDs might be amenable to negative selection by the b6 weakly neutralizing antibody, which recognizes these Env-NDs (see Fig 6 above). Env(-)Δ808-NDs were passed through a b6-Protein A-Sepharose column, and the Env-NDs in the flow-through fraction were analyzed by ELISA (Fig 9). Negative selection reduced the exposure of the b6 epitope, and minimally affected the exposure of the other epitopes examined. Thus, Env-NDs are amenable to counter-selection by antibodies.

### Incorporation of lipid adjuvants in Env-NDs

We evaluated whether Env-NDs could incorporate lipid adjuvants that might enhance immune responses to an Env immunogen formulated in NDs. Two lipid adjuvants, lipopolysaccharide (LPS) or lipoarabinomannan from M. smegmatis (LAM-MS), were studied [80,81]. Both lipid adjuvants included in the lipid mixture used for preparation of the Env-NDs could be detected by ELISA in the Env(-)Δ808-NDs purified by size-exclusion chromatography (S3 Fig). Thus, the inclusion of lipid adjuvants in Env-NDs is feasible and may be useful in the event that a nanodisc presentation of Env to the immune system is contemplated.

## Discussion

Metastability is essential for the function of HIV-1 Env trimers. The high-potential-energy conformation of the unliganded Env is converted by receptor binding to lower-energy conformations on the virus entry pathway [1,5–7,10,11,18,21,85]. Even in its unliganded state, the Env from a laboratory-adapted HIV-1 strain has been shown to sample multiple conformations [115]. Understanding the conformations of primary HIV-1 Env can facilitate approaches to treatment and prevention. For example, presenting the relevant conformations of Envs from transmitted/founder HIV-1 may be critical to the development of vaccines.

Interaction with the viral membrane greatly influences the conformation of the HIV-1 Env trimer. In currently available detailed structures of HIV-1 Env trimers, the membrane-proximal external region, transmembrane region and cytoplasmic tail of gp41 is either deleted or disordered [51–54,58]. Changes in these regions have been reported to alter the conformation of the rest of the HIV-1 Env trimer [59–62], underscoring the value of studying Env in a more natural membrane environment. Nanodiscs provide such an opportunity, while allowing purified Env to be studied *in vitro*.

The Env-NDs prepared in our study contain pure preparations of the Env precursor in a conformation very close to that of the cleavage-negative Envs expressed on a cell surface. We used glutaraldehyde crosslinking to stabilize the Env-NDs, which otherwise aggregated into large proteoliposome-like structures during storage at 4°C. Glutaraldehyde crosslinking has been shown to exert an effect on HIV-1 Env antigenicity similar to that associated with gp120-gp41 proteolytic cleavage [97]. The Env-NDs retain the epitopes for broadly neutralizing antibodies, whereas the epitopes for most weakly or non-neutralizing antibodies are well masked. This masking is more efficient in the Env-NDs than in other modalities examined, including Env-proteoliposomes.

The 4E10 and 35O22 neutralizing antibodies recognized the HIV-1_JR-FL_ Env(-) and Env(-)Δ808 glycoproteins in NDs very efficiently, even more so than HIV-1 Env-expressing cells. Thus, the membrane-proximal external region of gp41, which is recognized by the 4E10 antibody [100], must be accessible on the Env(-)-NDs and the Env(-)Δ808-NDs. Similarly, 35O22, an extremely potent broadly neutralizing antibody targeting the gp120:gp41 interface [99], exhibited efficient binding to Env-NDs and Env-liposomes relative to cell-associated or purified Envs, particularly after room temperature incubation. Like that of 4E10, the antigen-binding region of 35O22 may interact with the viral membrane to bind Env [53]. Indeed, we observed a low level of binding of the 4E10 and 35O22 antibodies to empty NDs. Although 35O22 was originally reported to bind cleaved Env preferentially [99], our study demonstrates that 35O22 can efficiently bind uncleaved Env under a variety of conditions. Further work on the binding of these antibodies to native Env trimers should provide additional insights into the structure and function of Env. Antibodies with a specificity similar to that of 4E10 and 35O22 are generated in some HIV-1-infected individuals, suggesting that Env(-)-NDs could be useful in eliciting such antibody responses in a vaccine context [99,116].

The antigenic profile of the HIV-1 Env-NDs exhibits some differences from that expected of the functional Env trimer. These differences arise in part from the uncleaved nature of the Env incorporated into the NDs. The proteolytic cleavage of gp120 and gp41 has been shown to influence the exposure of the epitopes for neutralizing and non-neutralizing antibodies on the Env trimer [94,95,97,99,117–130]. This effect is particularly pronounced on soluble forms of Env oligomers [94,95,117–120], but is also evident on membrane-anchored Envs [97,99,121–130]. For example, the weakly neutralizing b6 antibody directed against the gp120 CD4-binding site does not efficiently bind the cleaved HIV-1_JR-FL_ on virions. However, we show that the Env-NDs can be negatively selected to reduce the exposure of the b6 epitope. Env conformers that expose other Env epitopes that are deemed undesirable may be likewise removed from the Env-ND population. Ultimately, devising approaches to incorporate proteolytically cleaved HIV-1 Envs into nanodiscs may more effectively mask non-neutralizing antibody epitopes than the use of negative selection.

Other groups have recently succeeded in incorporating the HIV-1 Env in nanodiscs [73] or saposin-lipoprotein nanoparticles [66]. These nanoparticles, like our Env-NDs, retain the epitopes for the potent neutralizing antibodies. Frauenfeld *et al*. [66] showed that the Env in saposin-lipoprotein nanoparticles retained the epitope for a broadly neutralizing antibody even after prolonged 37°C incubation. Conversely, the CD4-induced epitope recognized by the 17b antibody was masked in this nanoparticle, even after the 37°C incubation. Although our Env-NDs were characterized with a more extensive array of antibodies, the results are consistent and suggest that detergent-solubilized and purified HIV-1 Env can assume a reasonably stable antigenic state when it is in a membrane.

Future studies will explore means of incorporating and studying fully functional mature HIV-1 Envs in NDs, and improving further the antigenic characteristics of the Env complexes.

## Supporting information

S1 Fig Screening detergents for HIV-1 Env purification. (A) The panel of detergents was tested for the ability to solubilize HIV-1_JR-FL_ Env(-)Δ808 glycoproteins expressed in CHO cells. The critical micelle concentrations (CMC) of each detergent is indicated. The resulting oligomeric state of the solubilized Env was judged by size-exclusion chromatography (SEC) and Western blotting of the SEC fractions. Detergents highlighted in green were used to solubilize the Env(-)Δ808 glycoprotein, which was then precipitated by a small panel of antibodies. (B) SEC fractions of the HIV-1_JR-FL_ Env(-)Δ808 glycoprotein solubilized in the indicated detergents were Western blotted to identify the positions of the Env(-)Δ808 glycoprotein complexes. The positions correspond approximately to the oligomeric states of Env as follows: 1—monomer; 2—dimer; 3—trimer; 4—higher-order oligomers; 5—aggregates near the void volume. Plotted curves represent the results of Western blotting SEC fractions, which were quantified for Env and plotted with ImageJ. (C) Representative Western blot of the Env(-)Δ808 glycoprotein solubilized in Brij-98 and precipitated by the indicated ligands. Ligands are colored to reflect expected binding to native, unliganded Env conformations (green) or a CD4-induced conformation (red). The supernatants were also Western blotted, using a rabbit anti-gp120 primary antibody (Abcam).
(PPTX)

S2 Fig Effects of free-thawing and denaturant on the antigenicity of Env-NDs subjected to incubation at room temperature for one week. Recognition on ELISA plates by the indicated ligands of HIV-1_JR-FL_ Env(-)Δ808-NDs that had been incubated at room temperature for one week, and then further freeze-thawed or treated with urea. For the first freeze-thaw cycle, only HIV-negative human serum and the ligands 2G12, 17b, 17b + sCD4, E51, 35O22 and 4E10 ligands were tested. * = p < 0.05, unpaired t-test. Potently neutralizing antibodies are highlighted in green, and weakly neutralizing antibodies in red. ND = Not determined.
(PPTX)

S3 Fig Lipid adjuvant incorporation into NDs. HIV-1_JR-FL_ Env(-)Δ808-NDs were assembled with brain lipids plus either lipopolysaccharide (LPS) or lipoarabinomannan from M. smegmatis (LAM-MS). LPS was used at a concentration of 1% of total lipids, and LAM-MS was used at 5 μg/ml final concentration. The Env(-)Δ808-NDs were captured on ELISA plates, which were incubated with antibodies against LAM-MS and LPS.
(PPTX)
