# Supplementary material for: Antigenic characterization of the human immunodeficiency virus (HIV-1) envelope glycoprotein precursor incorporated into nanodiscs
Source: PLoS One. 2017 Feb 2;12(2):e0170672. doi: 10.1371/journal.pone.0170672 (PMC5289478; doi:10.1371/journal.pone.0170672)
Supplement: S1 Fig — (A) The panel of detergents was tested for the ability to solubilize HIV-1JR-FL Env(-)Δ808 glycoproteins expressed in CHO cells. The critical micelle concentrations (CMC) of each detergent is indicated. The resulting oligomeric state of the solubilized Env was judged by size-exclusion chromatography (SEC) and Western blotting of the SEC fractions. Detergents highlighted in green were used to solubilize the Env(-)Δ808 glycoprotein, which was then precipitated by a small panel of antibodies. (B) SEC fractions of the HIV-1JR-FL Env(-)Δ808 glycoprotein solubilized in the indicated detergents were Western blotted to identify the positions of the Env(-)Δ808 glycoprotein complexes. The positions correspond approximately to the oligomeric states of Env as follows: 1—monomer; 2—dimer; 3—trimer; 4—higher-order oligomers; 5—aggregates near the void volume. Plotted curves represent the results of Western blotting SEC fractions, which were quantified for Env and plotted with ImageJ. (C) Representative Western blot of the Env(-)Δ808 glycoprotein solubilized in Brij-98 and precipitated by the indicated ligands. Ligands are colored to reflect expected binding to native, unliganded Env conformations (green) or a CD4-induced conformation (red). The supernatants were also Western blotted, using a rabbit anti-gp120 primary antibody (Abcam). (PPTX) [file pone.0170672.s001.pptx]

## Slide 1
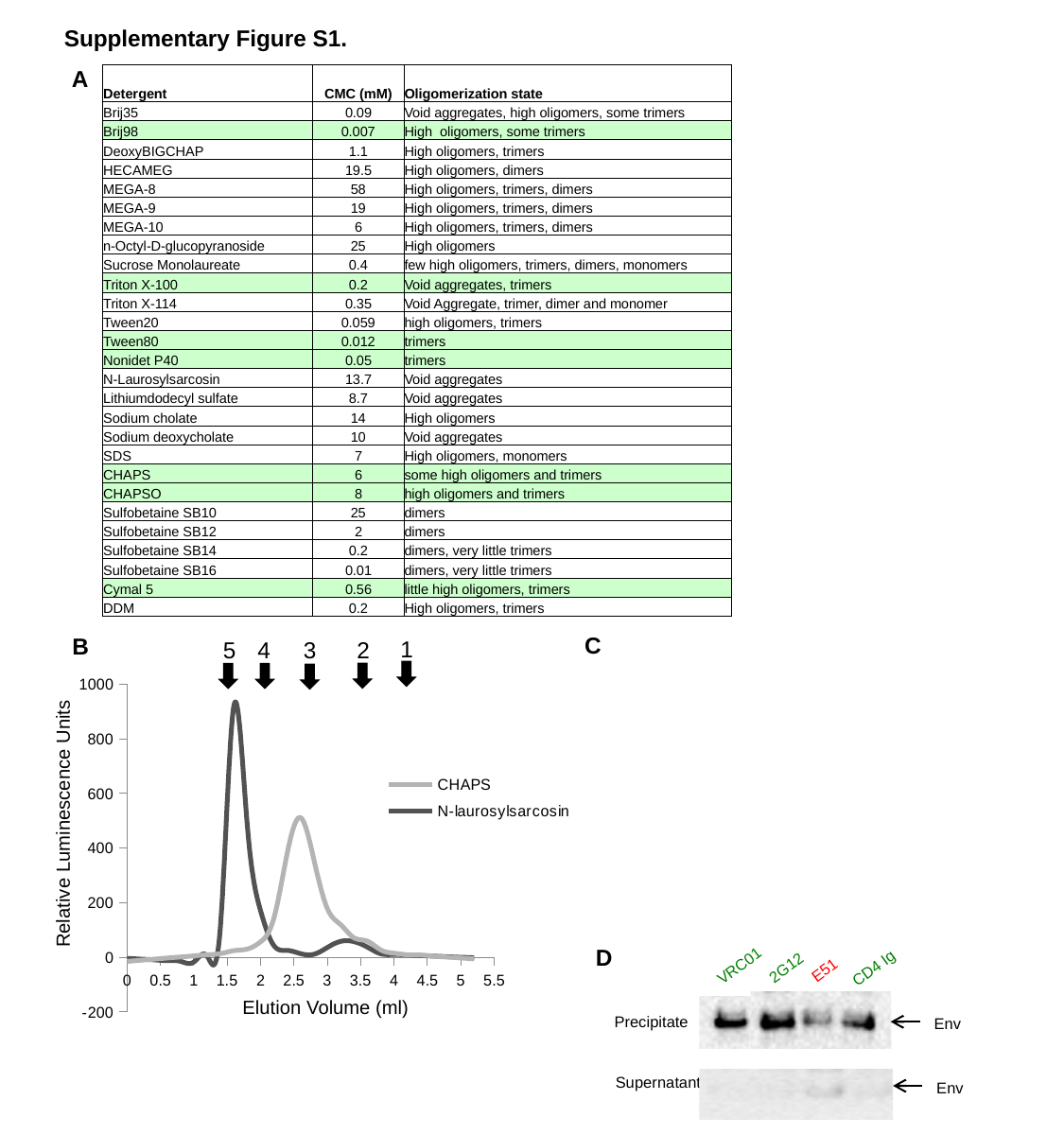

Supplementary Figure S1.
A
| Detergent | CMC (mM) | Oligomerization state |
| --- | --- | --- |
| Brij35 | 0.09 | Void aggregates, high oligomers, some trimers |
| Brij98 | 0.007 | High oligomers, some trimers |
| DeoxyBIGCHAP | 1.1 | High oligomers, trimers |
| HECAMEG | 19.5 | High oligomers, dimers |
| MEGA-8 | 58 | High oligomers, trimers, dimers |
| MEGA-9 | 19 | High oligomers, trimers, dimers |
| MEGA-10 | 6 | High oligomers, trimers, dimers |
| n-Octyl-D-glucopyranoside | 25 | High oligomers |
| Sucrose Monolaureate | 0.4 | few high oligomers, trimers, dimers, monomers |
| Triton X-100 | 0.2 | Void aggregates, trimers |
| Triton X-114 | 0.35 | Void Aggregate, trimer, dimer and monomer |
| Tween20 | 0.059 | high oligomers, trimers |
| Tween80 | 0.012 | trimers |
| Nonidet P40 | 0.05 | trimers |
| N-Laurosylsarcosin | 13.7 | Void aggregates |
| Lithiumdodecyl sulfate | 8.7 | Void aggregates |
| Sodium cholate | 14 | High oligomers |
| Sodium deoxycholate | 10 | Void aggregates |
| SDS | 7 | High oligomers, monomers |
| CHAPS | 6 | some high oligomers and trimers |
| CHAPSO | 8 | high oligomers and trimers |
| Sulfobetaine SB10 | 25 | dimers |
| Sulfobetaine SB12 | 2 | dimers |
| Sulfobetaine SB14 | 0.2 | dimers, very little trimers |
| Sulfobetaine SB16 | 0.01 | dimers, very little trimers |
| Cymal 5 | 0.56 | little high oligomers, trimers |
| DDM | 0.2 | High oligomers, trimers |
C
B
1
2
5
4
3
### Chart
| Category | CHAPS | N-laurosylsarcosin |
|---|---|---|Relative Luminescence Units
D
VRC01
2G12
CD4 Ig
E51
Precipitate
Env
Supernatant
Env
Elution Volume (ml)
