# Supplementary material for: Antigenic characterization of the human immunodeficiency virus (HIV-1) envelope glycoprotein precursor incorporated into nanodiscs
Source: PLoS One. 2017 Feb 2;12(2):e0170672. doi: 10.1371/journal.pone.0170672 (PMC5289478; doi:10.1371/journal.pone.0170672)
Supplement: S2 Fig — Recognition on ELISA plates by the indicated ligands of HIV-1JR-FL Env(-)Δ808-NDs that had been incubated at room temperature for one week, and then further freeze-thawed or treated with urea. For the first freeze-thaw cycle, only HIV-negative human serum and the ligands 2G12, 17b, 17b + sCD4, E51, 35O22 and 4E10 ligands were tested. * = p < 0.05, unpaired t-test. Potently neutralizing antibodies are highlighted in green, and weakly neutralizing antibodies in red. ND = Not determined. (PPTX) [file pone.0170672.s002.pptx]

## Slide 1
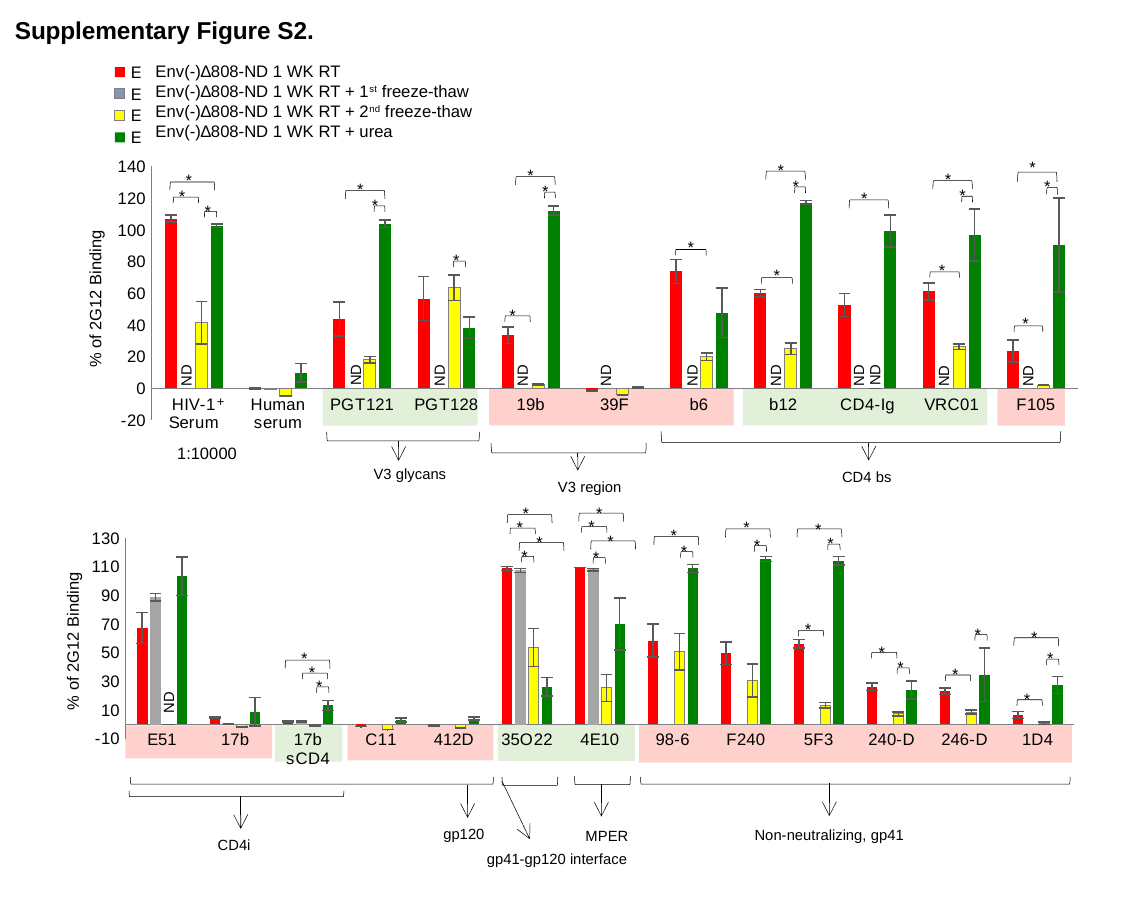

Supplementary Figure S2.
### Chart
| Category | Env ND | Env ND + first freeze thaw | Env ND + second freeze thaw | Env ND + urea |
|---|---|---|---|---|
| HIV-1 Serum | 107.04923699736129 | None | 41.370272412471074 | 102.55021179089935 |
| Human serum | 0.07063849867490157 | -0.8936511423739993 | -4.813944599656708 | 9.900930472377725 |
| PGT121 | 43.77810847659084 | None | 18.043469601415378 | 103.9411666004862 |
| PGT128 | 56.603703294046895 | None | 63.374673761721525 | 38.22581785054277 |
| 19b | 33.54260157027698 | None | 2.4784328610722253 | 112.0877055332704 |
| 39F | -1.6067803717387017 | None | -4.09523541478196 | 0.7917302993290093 |
| b6 | 73.72657933792125 | None | 19.95602155775819 | 47.65059300355866 |
| b12 | 59.950570378441405 | None | 24.901690437416022 | 117.12815636527813 |
| CD4-Ig | 52.656849378563 | None | 0.0 | 99.1446206389381 |
| VRC01 | 61.13161373159259 | None | 26.3777995135523 | 96.65341204058505 |
| F105 | 23.628808840266537 | None | 2.022351505068482 | 90.42278472112474 |Env(-)∆808-ND 1 WK RT
Env(-)∆808-ND 1 WK RT + 1st freeze-thaw
Env(-)∆808-ND 1 WK RT + 2nd freeze-thaw
Env(-)∆808-ND 1 WK RT + urea
*
*
*
*
*
*
*
*
*
*
*
*
*
*
*
*
*
*
% of 2G12 Binding
*
*
ND
ND
ND
ND
ND
ND
ND
ND
ND
ND
ND
+
V3 glycans
CD4 bs
V3 region
1:10000
*
*
*
*
*
*
*
### Chart
| Category | Env ND | Env ND + first freeze thaw | Env ND + second freeze thaw | Env ND + urea |
|---|---|---|---|---|
| E51 | 67.12315039617897 | 88.720192126845 | 0.0 | 103.32563523332192 |
| 17b | 4.592108876856521 | 0.03954274166549041 | -1.9306369427658927 | 8.741706593349285 |
| 17b sCD4 | 1.986133290565014 | 1.9575386037559181 | -1.122558089406015 | 13.206327699389206 |
| C11 | -1.5981743731266356 | None | -3.646130108 | 2.957234969023565 |
| 412D | -0.8905475812090017 | None | -2.6275679491463424 | 4.026314415775944 |
| 35O22 | 108.81570382603162 | 107.24906629361634 | 53.637805744514715 | 26.290142100076 |
| 4E10 | 109.4911457847498 | 107.85924957471795 | 25.353873409284365 | 69.92004604056757 |
| 98-6 | 58.40380096346736 | None | 50.60076114644081 | 108.75852305304312 |
| F240 | 49.64803487219933 | None | 30.53605387680879 | 115.20754595122392 |
| 5F3 | 56.227436327883765 | None | 13.347355783908888 | 114.02666120293873 |
| 240-D | 26.184732669670215 | None | 7.321981713365178 | 23.922045748314584 |
| 246-D | 23.03057641996804 | None | 8.467343870688625 | 34.686287324002954 |
| 1D4 | 6.572730362800577 | None | 0.6331792733885323 | 27.387374723531053 |*
*
*
*
*
*
*
*
% of 2G12 Binding
*
*
*
*
*
*
*
*
*
*
ND
gp120
Non-neutralizing, gp41
MPER
CD4i
gp41-gp120 interface
