# Supplementary material for: Antigenic characterization of the human immunodeficiency virus (HIV-1) envelope glycoprotein precursor incorporated into nanodiscs
Source: PLoS One. 2017 Feb 2;12(2):e0170672. doi: 10.1371/journal.pone.0170672 (PMC5289478; doi:10.1371/journal.pone.0170672)
Supplement: S3 Fig — HIV-1JR-FL Env(-)Δ808-NDs were assembled with brain lipids plus either lipopolysaccharide (LPS) or lipoarabinomannan from M. smegmatis (LAM-MS). LPS was used at a concentration of 1% of total lipids, and LAM-MS was used at 5 μg/ml final concentration. The Env(-)Δ808-NDs were captured on ELISA plates, which were incubated with antibodies against LAM-MS and LPS. (PPTX) [file pone.0170672.s003.pptx]

## Slide 1
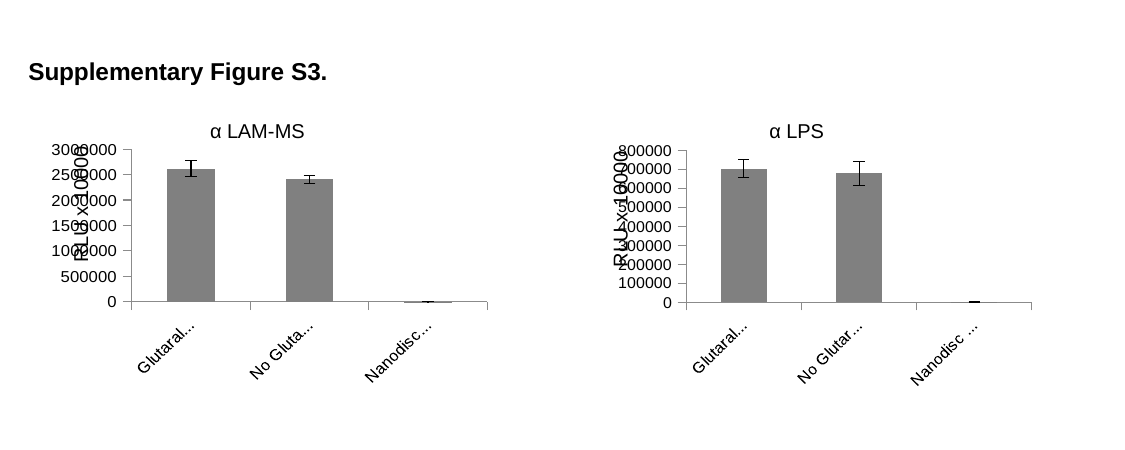

Supplementary Figure S3.
RLU x 10000
RLU x 10000
### Chart
| Category | anti LAM |
|---|---|
| Glutaraldehyde | 2613438.75 |
| No Glutaraldehyde | 2404917.6 |
| Nanodisc control | 1464.666666666667 |
### Chart
| Category | anti LPS |
|---|---|
| Glutaraldehyde | 702406.3333333334 |
| No Glutaraldehyde | 676835.666666667 |
| Nanodisc control | 391.3333333333333 |α LAM-MS
α LPS
